# Multiscale Viscoelastic Analysis of Asphalt Concrete

**DOI:** 10.3390/ma18245536

**Published:** 2025-12-10

**Authors:** Marek Klimczak

**Affiliations:** Faculty of Civil Engineering, Cracow University of Technology, Warszawska 24 Street, 31-155 Cracow, Poland; marek.klimczak@pk.edu.pl

**Keywords:** asphalt concrete, homogenization, representative volume element, viscoelasticity, Burgers model, finite element method

## Abstract

Numerical modeling of asphalt concrete and other asphalt mixes used in road engineering is an actively developed research field. In this study, a framework combining the following aspects is presented: (1) reliable reconstruction of the real samples; (2) using realistic material models of the microstructure constituents; and (3) providing high numerical efficiency. Asphalt concrete microstructure was reconstructed using image processing. The Burgers material model was applied to the subdomains identified as the mastic, and the linear elastic model was used for the aggregate particles. In order to increase the numerical efficiency, the developed homogenization method was used to accelerate the finite element analysis. The main novelty of this study is the integration of the Burgers material model with the numerical homogenization in the small strains range. A homogenization error measured in the maximum norm was smaller than 7% in the presented numerical examples (6.8% for the elasticity and 6.9% for the viscoelasticity problem, respectively). Simultaneously, the observed reduction in the number of degrees of freedom was larger than 510 times. The obtained results confirmed the applicability of the developed methodology to the analysis of the viscoelastic materials in the range of the small strains.

## 1. Introduction

The laboratory experiments and in situ tests are supplemented by the numerical analyses in the development of the novel materials that are used in civil engineering. A variety of professional equipment facilitates the observation of the lower scale phenomena. Thus, the understanding of the impact of the internal processes on the effective material response has rapidly increased.

In the context of the laboratory experiments, for instance, one can take advantage of the traditional testing machines that are equipped with a computed tomography (CT) [1,2,3]. As a result, one can obtain not only the overall specimen response in the test but also the results at the meso- or microresolution scale can be analyzed. This approach enables tracking the development of some phenomena (e.g., cracking [2,3]) ab initio, which is crucial in defining some threshold parameters in the constitutive material models. Moreover, the declared measurement accuracy of less than 1 µm makes it possible to track the response of the interface transition zone (ITZ) within the whole specimen. Several years ago, such studies referred to the samples of rigorously limited spatial dimensions. Scanning electron microscopy (SEM) [4], for instance, was able to capture such phenomenon but not for the whole asphalt mixture cores. Both methods (CT and SEM) are non-destructive in nature; however, typically the specimen is broken to some extent after the actual test.

In the case of the in situ tests, one can also investigate some phenomena not only in the traditional manner with the overall pavement response recording (e.g., deflections [5] or temperature maps [6,7]). In addition, some insight into the pavement structure is nowadays possible due to techniques such as ground penetrating radar (GPR) [8]. These methods are not destructive; thus, the measurements can be repeated for the same road segments when necessary.

It can be observed that the direction of the measurement devices’ development is towards more and more detailed scales. The most advantageous are the measurement techniques that are non-destructive in nature and those that allow for the analyses of the specimens with realistic dimensions. In other words, the simultaneous overall specimen response and its internal behavior tracking are of particular interest [1,2,3,7,8].

In the context of numerical modeling of asphalt concrete and other asphalt mixtures, the meso- and microresolution are also systematically studied in more detail. As far as the geometry is concerned, traditional approaches based on its idealization or substantial simplification are used for tests rather than for realistic analyses. Voronoi diagram-based techniques [9,10], the Monte Carlo method [11], grid partitioning [12], relocation of the regular mesh nodes [13] and other techniques are used for this purpose. They are primarily applied to the studies where the focus is on modeling aspects other than realistic geometrical representation or where the computational efficiency is of the highest priority.

It should be remarked that the trade-off between realistic internal structure recognition and numerical efficiency can however be obtained. It can be performed using, e.g., the error-controlled geometry simplification of the real structure (see [14]).

In the present state-of-the-art development, the method with the most established renown in the internal microstructure recognition is X-ray computed tomography (XRCT). Its applications are present also in reference to asphalt concrete and other asphalt mixture types [15,16,17,18,19,20]. Image processing techniques are used to identify (typically two, i.e., mastic and aggregate) asphalt mixture phases. When modeling the internal structure at the mesoscale, the threshold value of 2 mm is commonly used to classify the particle as the aggregate. Smaller fractions constitute the mastic phase. In some studies [17,19,20], the voids are also reconstructed to study the impact of their content on the behavior of the specimen. The superiority of the XRCT compared to other approaches (e.g., SEM [4]) is due to the fact that one can precisely reconstruct the meso- or microstructure within the whole specimen. It makes it feasible to investigate in detail (see, e.g., [1,2,3]) not only the geometry of the internal structure but also the localized phenomena within the real specimens.

After recognition of the meso- or microstructure, one can carry out additional numerical analyses to validate the proposed constitutive model or to identify its parameters. In terms of the finite element method, the transition from the XRCT to the geometry model can be the bottleneck of the whole approach. It is due to the fact that, typically, the voxel-based methods are used. Namely, each voxel is in a straightforward manner addressed as the finite element. When implementing such a transfer method, it is very natural and convenient to proceed in that mode. On the other hand, such an approach results in the finite element meshes of the extreme densities. In the case of the relatively large specimens and nonlinear analysis, it may lead to the numerical problem with prohibitively long computational time or even reach the limits of the computational resources. To some extent, this problem can be reduced by the error-controlled geometry simplification techniques [14]. But the evolving microstructure of some materials [21] can be generally an additional numerical challenge.

In order to overcome the limitations of the direct analysis of the asphalt mixture with the real microstructure recognized by some method of the image processing, various kinds of the multiscale upscaling or homogenization methods are used [13,22,23,24,25], to mention only a few. Their common idea is to carry out an effective macroscale analysis with incorporating the lower scale information. The aim is to obtain reliable results in a short time with the acceptable error introduced when comparing to the direct lower scale analysis.

In the above mentioned incomplete selection of homogenization methods used for the realistic microstructures, the group originating from the representative volume element (RVE) concept plays a vital role. RVE is defined as the small subdomain capturing all the necessary information on material heterogeneity. Its size has both the upper and lower bounds. RVE should be much smaller than “the characteristic dimension of the macroscale” and simultaneously much larger than “the characteristic dimension of the microscale”. One can find the studies where the proper RVE size itself is the main scientific problem [26,27]. In the case of asphalt mixtures, the upper bound is, in fact, specified by the layer thickness. Consequently, the lower bound is defined by the aggregate fraction sizes.

The most popular among RVE-based methods is computational homogenization (CH). Its applications to various materials and consecutive developments can be found in numerous scientific studies, e.g., in [28,29,30], to mention only a few. RVE is typically associated with a Gauss integration point. An iterative CH procedure consists of two main phases. Firstly, one solves a macroscale problem and computes deformation at a specific Gauss point. Then, this deformation is transferred to the RVE domain in the form of the boundary conditions. After solving this boundary value problem (BVP), one returns to the macroscale with the effective tangent matrix. These computations are typically iteratively repeated until the STOP conditions are fulfilled. It should be noted that the number of RVEs is equal to the number of Gauss points, which can be cumbersome in terms of computational time. A standard approach in CH implementation, thus, is parallelization. In transient nonlinear analysis, however, iterations at both scales are necessary.

In the proposed method, which is tailored to the small strains problems, the numerical homogenization (NH) is developed. In this concept, one solves a number of simple numerical tests to evaluate the effective material parameters [25,31,32]. As a result, one solves a macroscale problem with locally homogenized parameters.

In this study, the NH is used as a part of the developed framework that consists of three main aspects:

- Reliable recognition of the AC microstructure in 2D using the high-quality image processing;
- Carrying out a viscoelastic analysis with a Burgers material model used for the mastic phase;
- Facilitating the analysis with the RVE-based local evaluation of the effective parameter tensor.

The main aim of this study is to demonstrate a versatile methodology of the AC (and other asphalt mixtures) modeling in an efficient manner. The numerical tests confirmed its applicability. The obtained results are in very good agreement with the reference ones that were obtained using the extremely refined mesh to capture all the heterogeneities present within the analyzed specimen. The homogenization error does not exceed 5% for any of the tests. Taking into account that the reference solution was obtained using nearly 50,000 degrees of freedom and the corresponding homogenized solution was obtained using only 90 degrees of freedom, the methodology proved to be efficient.

Some aspects of this study have been developed in previous papers [13,14,25,33,34]. Particularly, the image-processing procedure with the integrated error-controlled geometry simplification techniques were presented in [14]. In this paper, those findings were used to reconstruct the real specimen microstructure of practically used dimensions. In [13,33], the Burgers material model was tested. Precisely, in [33] it was used for the domain with a digitally reconstructed microstructure according to the procedures developed in [14]. In the present paper, the finite element analysis carried out in the form demonstrated in [33] is used as the reference one. It provides reliable results but is computationally expensive. In order to facilitate the numerical analysis, numerical homogenization was used. Its higher order version with additional enhancements accounting for the heterogeneous RVE edges was developed in [25] for the linear elasticity problem. In the current study, this methodology was adjusted to the viscoelasticity problem in the small strains range. The main novelty of this paper consists of this particular part of the whole framework. In order to avoid exhaustive repetitions, the reader is referred to the previous papers and only their main ideas were recalled to make the presented framework coherent.

One can find a number of other approaches to the numerical modeling of asphalt mixtures in the literature, e.g., [35,36,37,38,39,40], and the references therein, to mention only a few.

## 2. Materials and Methods

### 2.1. AC Specimens and High-Quality Images

In order to obtain the asphalt concrete microstructure geometry for further numerical modeling purposes, a set of cylindrical specimens (with initial height of 150 mm and the diameter equal to 150 mm) were prepared in the laboratory of the Chair of Highway, Railway and Traffic Engineering of the Civil Engineering Faculty (Cracow University of Technology, Cracow, Poland) as described in [37]. In this case, AC 16 of the typical composition was used, i.e., only a neat bitumen 35/50 (without any modifiers) and dolomite aggregate. These specimens were further used for a destructive semi-circular bending test. A subset of them was sliced with a circular saw to create 50 mm-high specimens for the microstructure recognition.

After dusting off the specimens and the proper preparation of the camera post (see [14] for details), the high-quality images of resolution 24 Mpx were taken. For the purposes of numerical modeling, some trimming operations were performed to create rectangular shapes of the specimens. In this study, a comparison between two numerical approaches (direct analysis and numerical homogenization) is mainly presented. Thus, the obtained specimens should be understood as the virtual ones. There is no corresponding laboratory experiment to the numerical tests. A set of complimentary physical material parameters can be found in [34]. There is no analysis of the relationship between the measured physical parameters and the Burgers model parameters. The latter ones were taken from the literature. The model validation is also beyond the scope of this study. Thus, the only input data required for the potential repetition of the numerical tests is the high-quality image or even the reconstructed microstructure itself.

### 2.2. Microstructure Geometry Recognition

In Section 1, a number of approaches to the recognition of the underlying structure were presented. They have their own advantages and disadvantages that should be accounted for when selecting a reliable method. In this study, the approach based on image processing was selected. Due to the restriction of the modeling space to 2D, the high-quality images are sufficient in the process of internal structure recognition. Practically, we use a threshold of 2 mm in reconstruction of the aggregate particles. Thus, two phases are modeled, i.e., a mastic (a mixture of neat bitumen, filler and an extremely fine aggregate) and aggregate. For the sake of convenience, the internal structure at this scale of observation is called the microstructure within this paper. Consequently, the numerically homogenized specimen is analyzed at the scale called the macroscale. It should be remarked that the mesoscale/mesostructure terms are used in the context of the scale of the observation associated with the asphalt mixture in some papers [9,10,11,12]. In those approaches, the microstructure refers to the scale of micrometers. At this scale, the interfacial transition zone can be investigated, which is beyond the scope of the present paper.

**Figure 1:**
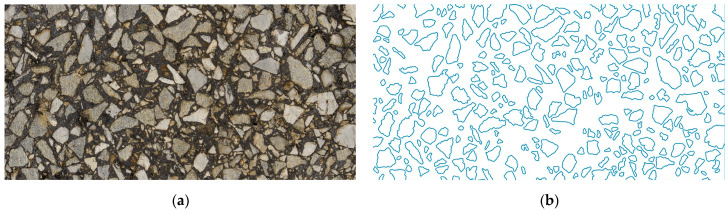
Analyzed asphalt concrete specimen: (**a**) high-quality image after trimming operation; (**b**) reconstructed microstructure geometry.

In Figure 1a, the high-quality image of the analyzed AC specimen is presented. In Figure 1b, the corresponding microstructure geometry is shown.

In Figure 1, the effect of the standard image processing procedure [14,25] is presented. Since the main focus of this paper is on the homogenization procedure, the steps of the image processing are only briefly recapitulated below, and the reader is referred to [14] for details:

- Taking of the high-quality images with the specimens dusted off, cleaned and light adjusted properly in order to eliminate any factors that can potentially reduce the quality of the image;
- Converting truecolor images to the grayscale based on the intensity (see [14] for details on the intensity function used);
- Adaptive binarization of the grayscale image (with the sensitivity parameter set as 0.65 in this study);
- Removing holes, filtering (removing inclusions below the 2 mm threshold) and other minor processing (e.g., erosion) to obtain the binary image of the biphasic domain;
- Segmentation and boundary of each aggregate particle detection;
- Optionally, some processing of the boundary shape. Herein, the error-controlled algorithm developed in [14] was used to simplify the description of the microstructure geometry. Practically, up to five iterations were used for every inclusion due to the selected precision in area reconstruction equal to 10%. As demonstrated in [14], this simplification is justified at this scale of analysis, and it introduces only negligible error to the solution. Simultaneously, the reduction in the finite element mesh density is substantial, which reduces the computational cost of the whole framework.

### 2.3. Constitutive Modeling

In this paper, the analysis of asphalt concrete response is restricted to the 2D problem. The plane strain state is consequently assumed. Another restriction of the modeling procedure is the small strains range of analysis. This assumption is in line with the values observed in the mechanistic-empirical approach [35], where the response after a single tire load impulse is measured.

As a result of the procedures presented in Section 2.2, one obtains biphasic (mastic and aggregate) domain geometry. We follow a typical assumption on the linear elastic behavior of dolomite aggregate particles [36,37]. For the mastic phase, a well-established Burgers material model [36,37] is used. Since it is a typical model of the linear viscoelasticity used for the bituminous materials, we refer to [36,37] for its detailed description. Below, some aspects of this model, which are important for the numerical homogenization step, are summarized:

- Generalized Burgers material model comprises a number of Kelvin–Voigt and Maxwell elements linked in series;
- Consequently, the total strain ε can be additively decomposed into elastic $\varepsilon_{EL}$, viscous $\varepsilon_{V}\;$ and viscoelastic term $\varepsilon_{VEL}$;
- Generally, the moduli are associated with the springs in the mechanical interpretation of the model; thus, they represent the elastic material behavior. The viscosities are associated with the dumpers in the mechanical representation; they model the viscous behavior of the material. Specifically, the instantaneous and recoverable material response is modeled by the Maxwell element’s spring. The irrecoverable material response is modeled by the dumper of this element. The delayed, yet recoverable, material response is modeled by the Kelvin–Voigt element (parallel combination of the spring and dumper).
- In the absence of the body forces, linearized incremental formulation of the viscoelasticity problem yields the following:

Find the vector field of displacement increments $\Delta u\left(x,t\right)\in H_{0}^{1}\left(\Omega \right)+\Delta \hat{u}(x,t)$ such that

(1) $$\int_{\Omega }\varepsilon \left(v\right):C^{-1}\Delta \varepsilon \left(\Delta u\right)d\omega =\int_{S_{\sigma }}v\cdot \Delta \hat{t}ds+\int_{\Omega }\varepsilon \left(v\right):C^{-1}\Delta \varepsilon^{*}d\omega \;\;\;\;\forall v\in H_{0}^{1}\left(\Omega \right)$$

where t stands for time, x is the point position, C is the material parameter tensor, $\varepsilon^{*}$ is the inelastic strain tensor, Ω is the whole analyzed domain, $S_{\sigma }$ stands for the boundary part with the Neumann boundary conditions, v stands for a test function, $\hat{t}$ are the applied tractions (external load), $\hat{u}$ are the known displacements and $H_{0}^{1}$ is the Sobolev space of functions satisfying homogeneous Dirichlet boundary conditions.

In the small strain range and transient analysis, we make use of this formulation to eliminate the necessity of the tangent matrix assessment at every time step. Instead, one proceeds with one stiffness matrix (see the left-hand side of Equation (1)) and updates only the contribution to the right-hand side due to the presence of the inelastic strain tensor $\varepsilon^{*}$ (sum of the viscous and viscoelastic terms) that are defined as [35]

(2) $$\Delta \varepsilon_{VEL}=\sum_{i=1}^{N}\left\{\varepsilon_{VEL,t-\Delta t}^{i}\left(e^{\frac{-\Delta t}{\tau_{i}}}-1\right)+\Delta te^{\frac{-\Delta t}{2\tau_{i}}}C_{VEL}^{i}\left(\sigma_{t-\Delta t}+\frac{\Delta \sigma }{2}\right)\right\}$$

(3) $$\Delta \varepsilon_{V}=\Delta tC_{V}\left(\sigma_{t-\Delta t}+\frac{\Delta \sigma }{2}\right)$$

where σ is the stress tensor, t−Δt denotes the previous time instance, N denotes the number of the Kelvin–Voigt elements representing viscoelastic material behavior with $\tau_{i}$ being their retardation times, and $C_{V}$ and $C_{VEL}$ are constitutive matrices of the respective inelastic elements.

The workflow of the numerical analysis, in the form we use it, is presented in [33]. For the sake of clarity, its main aspects are recalled below:

- The analysis is carried out with a division of the whole analysis period into a set of properly selected time instances—discretization of the time domain with intervals Δt;
- The initial solution increment at every time instance is the elastic solution increment; it is equivalent to the solution of Equation (1) without its rightmost term;
- Inelastic strain increments are computed according to Equations (2) and (3);
- The load vector contributions (the rightmost term in Equation (1)) are computed elementwise and assembled;
- Equation (1) is solved in its full form;
- Iterative procedure is repeated to obtain the equilibrium;
- Final incremental quantities of displacements, strains and stresses are saved and the response at the next time instance is evaluated.

In typical implementations, the tangent stiffness matrix (left-hand side) is updated at each time instance. The right-hand side consists only of the external load contribution. In the approach demonstrated by Equation (1), we can proceed in a different way in the small strains range. Due to the additive decomposition of total strains, their inelastic term is put to the right-hand side. Illustratively, the evolution of the inelastic strains (contributing to the load vector) is used instead of the evolution of the material parameter tensor (modifying the left-hand side).

### 2.4. Numerical Homogenization of the Burgers Material

- In this paper, we extend the findings of the study [25] in the context of the viscoelastic analysis in the small strains range. Herein, we use only the linear approximation in the finite element analysis and do not modify the RVE boundary condition, which was the main aspect of that paper.
- Generally, the idea of the numerical homogenization is to solve a set of simple numerical tests (see Section 3)—in 2D: tensile and shear tests in both directions [25]—and using the average theorem to compute the average strain $<\varepsilon_{ij}>$ and stress $<\sigma_{ij}>$ tensor components (Equations (4) and (5)) within the RVE:
  (4) $$<\varepsilon_{ij}>=\frac{1}{V}\int_{\Omega }\varepsilon_{ij}d\Omega$$
  (5) $$<\sigma_{ij}>=\frac{1}{V}\int_{\Omega }\sigma_{ij}d\Omega .$$

Subsequently, these quantities are used to compute the effective tensor of material parameters from Equation (6):

(6) $$<\sigma >=C_{eff}<\varepsilon >$$

In the tests performed in this study, we assumed that the effective tensors are anisotropic, which is the most general approach. The assumption on the isotropy is not valid due to the non-periodic microstructure within the RVE’s.

In terms of the viscoelastic analysis, it would be generally necessary to use the above procedure for each time instance. However, using the formulation described by Equation (1), we can evaluate effective tensors of material parameters only once within the whole analysis and update only the inelastic strain increments at Gauss points.

Practically, we split the analysis of the inelastic behavior into the Gauss points and their corresponding RVEs, which is very profitable due to the possibility of parallelization.

The general procedure of the numerical homogenization for the Burgers material can be summarized as follows:

- In the “offline” step, evaluate the effective tensors of material properties for every RVE associated with a respective Gauss point—this is performed only once;
- In the “online” step, solve the macroscale problem at a specific time instance and compute the strains at every Gauss point;
- Use the Gauss points’ strains to impose the kinematic boundary conditions for the corresponding RVEs (u=ε×x, where u denotes the displacements along the boundary, ε is the average stress tensor within the macro element and x stands for the point position);
- Solve these local BVPs using Equation (1) (for a given time instance) and compute average inelastic strains;
- Use these inelastic strains to update the load vector in Equation (1) at the macroscale level.

It should be noted that the online step is the main computational cost of the whole transient analysis, since the iterative procedures are present at both levels of analyses, i.e., at the micro- (RVE level) and macroscale (whole specimen level). The benefit of the developed approach is the independent split of the viscoelastic analysis to the subdomains corresponding to specific RVEs.

## 3. Results

**Figure 2:**
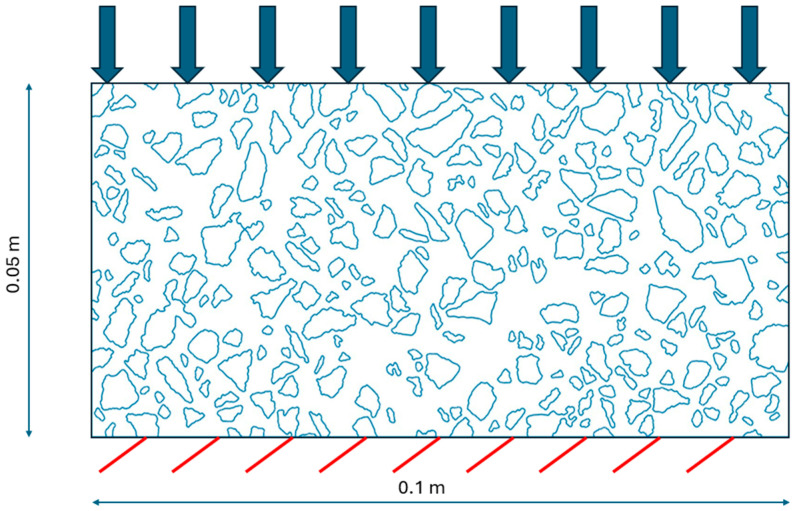
Analyzed domain with a digitally reconstructed microstructure and boundary conditions.

**Figure 3:**
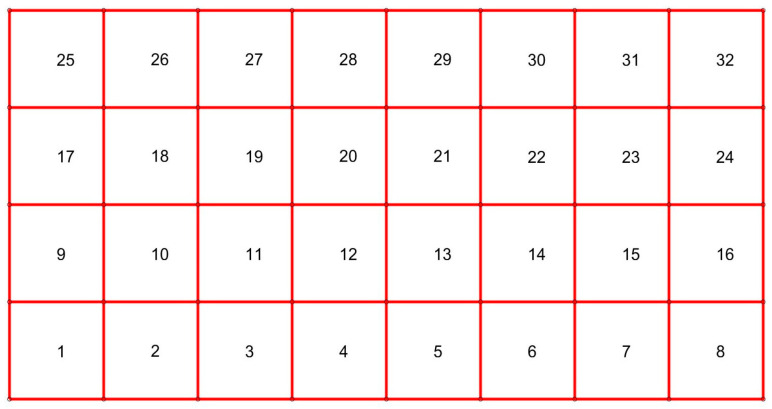
Coarse mesh used for the solution at the macroscale level.

In this section, the application of the presented framework is numerically tested. We begin with the initial test of the linear elasticity to illustrate some of the numerical aspects (Section 3.1). Subsequently, the main test shows the results of the full viscoelastic analysis (Section 3.2). For both tests, the rectangular domain (see Figure 2) of dimensions 0.1 m × 0.05 m is analyzed. Its bottom edge is fixed, and the top edge is subject to the constant load of intensity equal to 80 kN/m. In both tests, we use an extremely refined mesh consisting of 23,680 nodes (47,360 degrees of freedom). Linear triangular elements are used for the solution approximation. For the sake of clarity, this mesh is not shown. The finite elements would not be visible globally at this scale of presentation. Instead, we show the coarse mesh (Figure 3) that was used to solve both tests at the macroscale. Linear quadrilateral finite elements were used at this scale of analysis. Their size was selected on the basis of the study [25] to fulfill the requirements for the RVE size. Practically, the whole coarse element is considered as the RVE. For the RVE boundary conditions, the average of 9 Gauss point strain values within the coarse mesh element are used. This approach was used to provide a comparison between a direct and homogenized solution obtained using precisely the same microstructure. Generally, it is not necessary. We used it for illustrative purposes.

**Figure 4:**
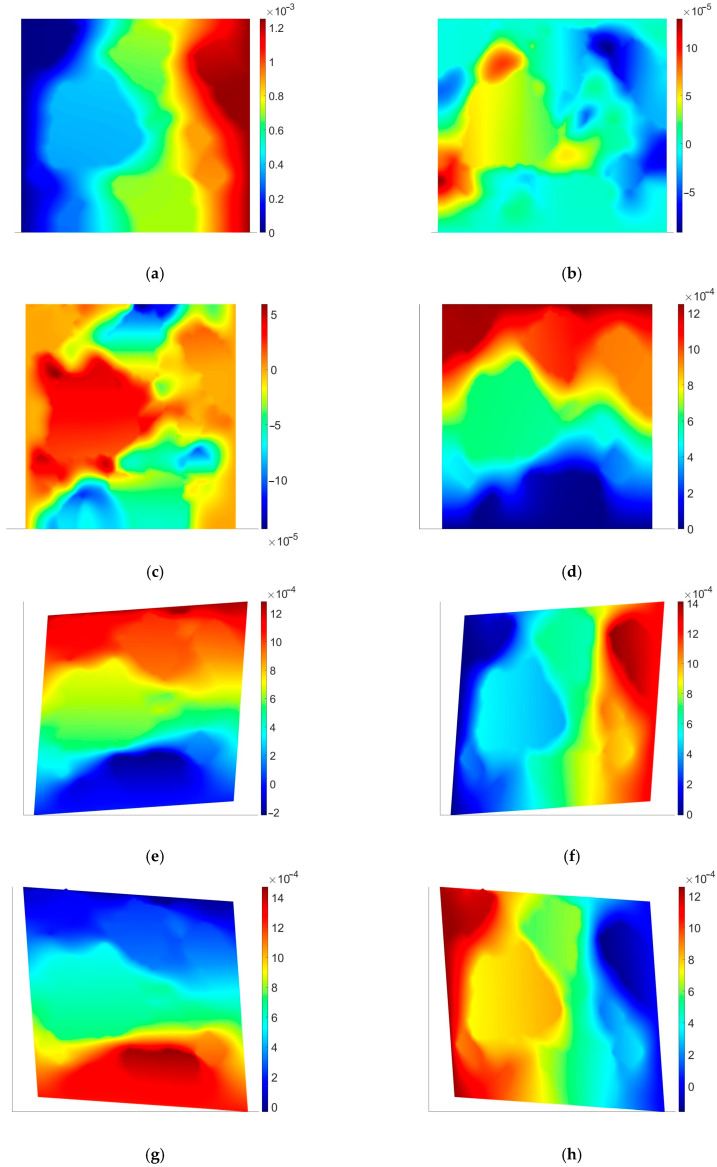
RVE associated with Gauss points in element no. 1; displacements: (**a**) *u_x_* for test 1; (**b**) *u_y_* for test 1; (**c**) *u_x_* for test 2; (**d**) *u_y_* for test 2; (**e**) *u_x_* for test 3; (**f**) *u_y_* for test 3; (**g**) *u_x_* for test 4; (**h**) *u_y_* for test 4. The results are presented in meters. Test numbers denote the following: 1—tension in horizontal direction, 2—tension in vertical direction, 3—shear in vertical direction, 4—shear in horizontal direction.

The effective coarse element tensors of material parameters were obtained using four simple numerical tests, i.e., tension and shear tests in both directions. For the sake of brevity, the results are shown only for coarse element no. 1 (see Figure 4).

One can easily observe in Figure 4 the solution fluctuations due to the heterogeneous microstructure. The remaining RVEs exhibit the same character, thus, they were skipped in the paper.

In Table 1, the linear elastic material properties are shown for the aggregate phase. In Table 2, mastic characteristics are shown, respectively. They were taken from the literature; no identification procedure was implemented within this study. Generally, material parameters of bitumen are highly dependent on temperature. In laboratory experiments, however, a constant temperature needs to be maintained. Thus, the same assumption was made on the numerical tests. Beyond reliable, yet computationally expensive, approaches based on field coupling (e.g., displacements and temperature), there is also another possibility. It originates from the observation that the responses of bitumen at various temperatures are of the same character. Quantitatively, this difference is represented by the so-called shift factors.

### 3.1. Linear Elastic Test

**Figure 5:**
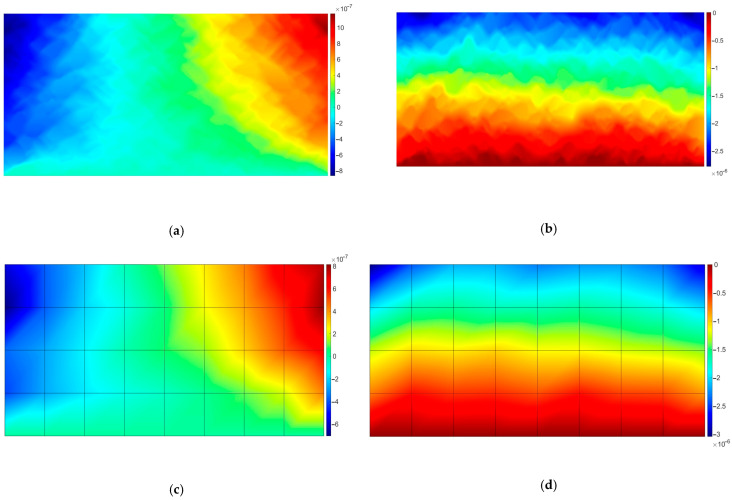
Comparison of the linear elastic reference and the homogenized solutions; displacements: (**a**) *u_x_* for the fine mesh; (**b**) *u_y_* for the fine mesh; (**c**) *u_x_* for the coarse mesh; (**d**) *u_y_* for the coarse mesh. The results are presented in meters.

In this test, the fine mesh reference solution is compared with the one obtained using the numerical homogenization (see Figure 5).

It can be easily observed that the reference solution exhibits many more local fluctuations than the homogenized one. It should be underlined that the approximation at the macroscale level is linear. The only quantity that “informs” about the underlying heterogeneous microstructure is the effective tensor of material parameters.

The observed reduction in the number of degrees of freedom (NDOF) exceeds in both tests 510 times. In terms of accuracy, the homogenization error measured in the maximum norm for displacements is approximately equal to 6.8%.

### 3.2. Viscoelastic Test

**Figure 6:**
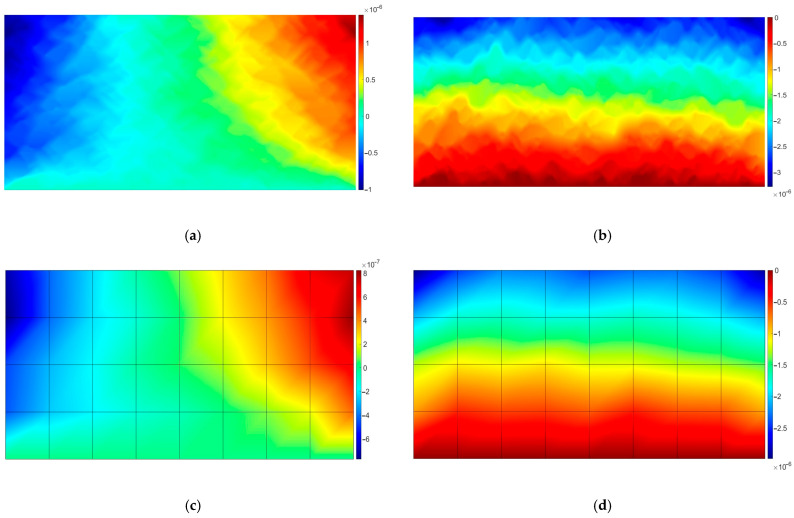
Comparison of the viscoelastic reference and the homogenized solutions; displacements: (**a**) *u_x_* for the fine mesh; (**b**) *u_y_* for the fine mesh; (**c**) *u_x_* for the coarse mesh; (**d**) *u_y_* for the coarse mesh. The results are presented in meters.

In this test, we extend the analysis to the viscoelasticity problem. The difference between the present and previous test is the fact that the mastic phase behaves as the Burgers material, which is in good agreement with laboratory experiments [34,35]. In this test, we simulate a single load impulse of duration equal to 0.2 s. Even such a short period of loading changes the response of the specimen (compare Figure 5 and Figure 6). A dominant vertical component exhibits maximum value observed in Figure 6, which is about 8.3% larger compared to the corresponding value observed in Figure 5.

**Figure 7:**
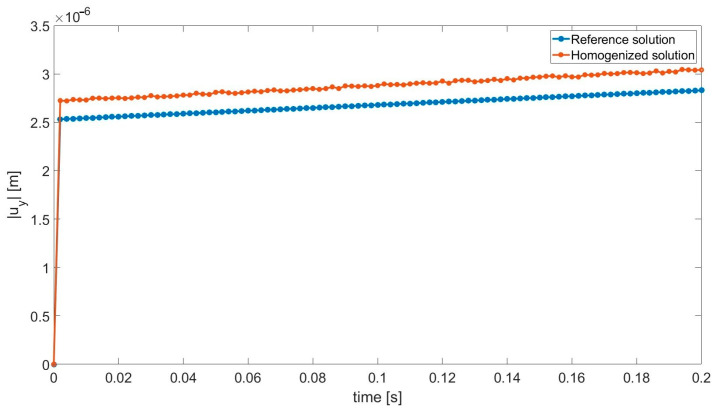
Absolute value of the vertical displacement in the function of time.

Due to a very short analysis period, one cannot visually observe the effect of the viscoelastic matrix (bitumen) in Figure 6, which presents the final specimen response. It can be studied in Figure 7, where the displacement of the selected point is shown.

The maximum local error is equal approximately to 6.9% in this test. This demonstrates the applicability of the proposed framework to the multiscale analysis of asphalt concrete and other asphalt mixtures. The large difference in the Young moduli of both constituents should be noticed.

In order to present the applicability of the proposed approach to the viscoelastic analysis, the history plot (Figure 7) is presented for a selected point with coordinates (0.1 m, 0.05 m). This is the top-right corner of the specimen. One can observe that after instantaneous elastic response, the creep phenomenon takes place. The load remains at the same level, whereas the displacement increases. Due to a very short analysis period in this test, no nonlinear behavior can be visually noticed. In longer analyses (see [13,36,37]), this effect can be easily observed.

**Figure 8:**
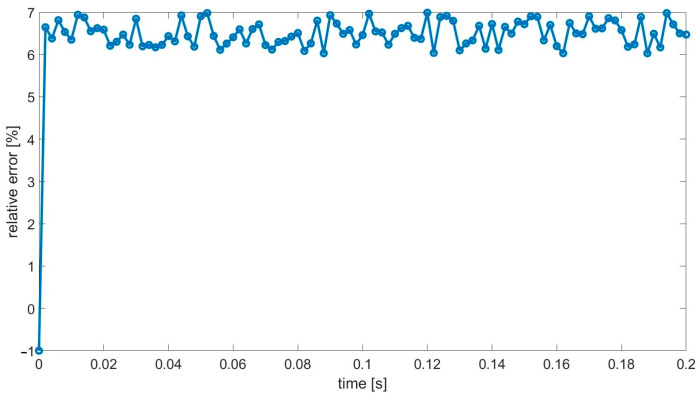
Relative error in the function of time.

Another interesting observation can be made looking at Figure 8, where the corresponding error history plot is presented. By error, we mean the absolute value of the difference between the presented solutions at each time instance divided by the absolute value of the reference solution at the corresponding time instance. One can observe that the error oscillates; however, there is no accumulation of its value in time. It is a promising result, which confirms the stability of the developed approach.

Future research effort is to provide additional enhancements to the methodology to reduce the homogenization error. However, a substantial reduction in the NDOF (more than 500 times in the above tests) should be noticed. The approximate relative error equal to 6.5% for each time instance seems acceptable in most practical applications.

In all tests and scales analyzed in this paper, only the linear approximation was used. The obtained results can be in better agreement with the reference ones when the approximation order at the macroscale is increased. Technically, there would be no necessity to repeat the offline step of the proposed framework, since the material parameter tensor does not depend on the approximation order.

In order to roughly estimate the correctness of the effective tensors of material parameter evaluation, a simple test is presented below. We assumed that at this scale of analysis, both the matrix and the inclusions themselves are homogeneous. Thus, their corresponding tensors of material parameters ($C_{matrix}$ and $C_{inclusion}$) are as follows:

$$C_{matrix}=10^{8}\left[\begin{array}{ccc} 9.4231 & 4.0385 & 0\\ 4.0385 & 9.4231 & 0\\ 0 & 0 & 2.6923 \end{array}\right]\mathrm{Pa}$$

$$C_{inclusion}=10^{10}\left[\begin{array}{ccc} 9.4231 & 4.0385 & 0\\ 4.0385 & 9.4231 & 0\\ 0 & 0 & 2.6923 \end{array}\right]\mathrm{Pa}$$

For element no. 1 (see Figure 4), the effective tensor of material parameters $C_{eff}^{1}$ evaluated using the numerical homogenization (see Section 2.4) is given below:

$$C_{eff}^{1}=10^{10}\left[\begin{array}{ccc} 2.0269 & 0.8108 & 0.1171\\ 0.8108 & 2.3528 & 0.1950\\ 0.1171 & 0.1950 & 1.2216 \end{array}\right]\mathrm{Pa}$$

Using a simple mixture rule for the same element (and assuming implicitly the periodic distribution of the inclusions), one obtains

$$C_{mix}^{1}=10^{10}\left[\begin{array}{ccc} 2.7063 & 1.1598 & 0\\ 1.1598 & 2.7063 & 0\\ 0 & 0 & 0.7732 \end{array}\right]\mathrm{Pa}$$

In the absence of laboratory experiment results, such a rough approximation can serve as the initial verification of the offline step in the whole framework.

## 4. Discussion

The obtained results demonstrated the applicability of the proposed framework. In this study, both the dimensions and the microstructure of the specimen were realistic. It should be remarked that it can be very challenging for other homogenization methods due to several cumbersome aspects:

- The microstructure geometry is very irregular; it does not exhibit periodicity;
- The dimensions of the specimens (corresponding to the thicknesses of the pavement layers) make it difficult to easily fulfill the requirement of the scale separation in context of the RVE size—in the numerical tests presented in this paper, the findings of [25] were used to specify the optimal RVE size;
- The value of the main parameter (the Young modulus) exhibits a very large difference between the phases of the specimen.

In the context of the aforementioned aspects, the developed numerical homogenization scheme demonstrated its high effectiveness. Recalling the main results presented in Section 3, several observations should be stressed:

- The relative homogenization error measured in the maximum norm was equal to 6.8% and 6.9% for the elasticity and viscoelasticity problem, respectively;
- The reduction in the NDOF is equal to about 510 times—it is particularly promising in the context of viscoelastic analysis;
- The relative error seems not to accumulate drastically—in the presented results, it oscillated around approximately 6.5% for the selected point;
- The effective tensor of material parameters is in line with a rough approximation using a simple mixture rule.

Further enhancement of the proposed framework can be obtained, e.g., by the increasing of the approximation order at the macroscale level, which was tested only for the linear elasticity problem [25]. The framework should also be tested for other constitutive models (see, e.g., [38,39,40]) and extended to a full 3D space. The microstructure would have to be recognized using the processing of the XRCT scans to generate a realistic geometry.

## 5. Conclusions

Concluding remarks are as follows:

- Image processing is a versatile tool for microstructure recognition;
- Multiscale analysis of asphalt concrete and other asphalt mixtures is necessary to investigate the microscale phenomena efficiently;
- Numerical homogenization in the presented form can be an effective method of viscoelastic analysis in the small strain range—it is the main novelty of the paper;
- Special attention should be paid to the accurate selection of the RVE size in such an analysis;
- Further research efforts should consider 3D analysis using the proposed framework with the microstructure recognized using the XRCT scans.

## Figures and Tables

**Table 1:** Material data for aggregate.

Property	Value	Unit
Young modulus E	70,000	[MPa]
Poisson ratio ν	0.3	[-]

**Table 2:** Material data for the mastic phase.

Property	Value	Unit
Young modulus E_M_^1^	700	[MPa]
Young modulus E_KV_^2^	120	[MPa]
Poisson ratio ν	0.3	[-]
Poisson ratio equivalent ν_M_^1^	0.3	[-]
Poisson ratio equivalent ν_KV_^2^	0.3	[-]
Viscosity η_M_^1^	60,000	[MPa s]
Viscosity η_KV_^2^	600	[MPa s]

^1^ Quantity referring to the Maxwell element in the Burgers model. ^2^ Quantity referring to the Kelvin–Voigt element in the Burgers model.

## Data Availability

The original contributions presented in this study are included in the article material. Further inquiries can be directed to the corresponding author.

## Abbreviations

The following abbreviations are used in this manuscript:

AC	Asphalt concrete
BVP	Boundary value problem
CH	Computational homogenization
CT	Computed tomography
FEM	Finite element method
GPR	Ground penetrating radar
ITZ	Interface transition zone
NDOF	Number of degrees of freedom
NH	Numerical homogenization
RVE	Representative volume element
SEM	Scanning electron microscopy
XRCT	X-ray computed tomography
